# Heart rate variability and amplitude-integrated electroencephalography measured shortly after birth and time to reach clinical milestones: a pilot study in late preterm infants

**DOI:** 10.3389/fped.2025.1579197

**Published:** 2025-06-05

**Authors:** Birju A. Shah, Samantha Latremouille, Sanjay Chawla, Martin Keszler, Richard Tucker, Abbot Laptook, Guilherme M. Sant'anna

**Affiliations:** ^1^ Department of Pediatrics, University of Oklahoma Health College of Medicine, Oklahoma City, OK, United States; ^2^ Department of Pediatrics, McGill University Health Centre, Montreal, QC, Canada; ^3^ Department of Pediatrics, Wayne State University, Detroit, MI, United States; ^4^ Warren Alpert Medical School, Brown University, Providence, RI, United States

**Keywords:** biomarkers, neurophysiological assessment, length of hospital admission, developmental maturation, clinical milestones, neonatal morbidities, late preterm babies

## Abstract

**Background:**

Among late preterm (LPT) infants, there is significant variability in reaching milestones for safe discharge. We examined the associations of early measures of heart rate variability (HRV) and amplitude-integrated electroencephalogram (aEEG) with time to wean to an open-air cot and to achieve full oral feeds.

**Methods:**

This is a prospective, multicenter observational cohort study that enrolled infants between 34^0/7^ and 34^6/7^ weeks gestational age (GA). Infants with growth restriction and major congenital anomalies were excluded. Electrocardiogram (ECG) for 1 h and cross-cerebral aEEG for 6 h were recorded within 96 h after birth. Correlations of HRV and aEEG parameters with outcomes were evaluated using stepwise linear regression.

**Results:**

Of the 26 infants from three centers, 23 were included for analysis for time to an open-air cot. The analysis for time to full oral feeds was limited to 19 infants from two centers with similar feeding policies. Including HRV parameters (*time domain*, median and standard deviation of *R*-wave to *R*-wave interval; *frequency domain*, ratio of the low frequency to high frequency power and their interaction) and aEEG parameters (total and immature *cycles*/hour) strengthened associations with time to open-air cot (adjusted *R*^2^ = 0.72) and time to full oral feeds (adjusted *R*^2^ = 0.53) compared with each parameter alone.

**Conclusions:**

Early measurements of HRV and aEEG parameters correlate with time to an open-air cot and to achieve full oral feeds in LPT infants born between 34^0/7^ and 34^6/7^ weeks GA.

## Summary highlights

• Among late preterm (LPT) infants, there is large variability in the time to reach milestones needed for discharge that cannot be explained by their gestational age (GA) alone.
• Early measurements of heart rate variability (HRV) and amplitude-integrated electroencephalogram (aEEG) parameters correlated with time to an open-air cot and to achieve full oral feeds in this prospective cohort of LPT infants born between 34^0/7^ and 34^6/7^ weeks GA.
• Differences in autonomic and central nervous system function may contribute to the large variability in the time to reach milestones for safe discharge among infants at the same GA.
• Both physiological measures of HRV and aEEG may serve as potential biomarkers of clinical trajectories and assessment of neurophysiological maturation.

## Introduction

Over the last 25 years, the overall rate of preterm birth has progressively risen. Much of this increase is due to late preterm (LPT) births, including infants born at a gestational age (GA) between 34^0/7^ and 34^6/7^ weeks (1, 2). Safe hospital discharge of preterm infants typically requires that respiratory morbidities, including apnea of prematurity, are resolved, core body temperature can be maintained at ambient temperature, and adequate feedings are taken orally (3). However, among LPT infants, there is large variability in the time to reach such milestones that can neither be explained by their GA alone nor by morbidities of prematurity (4, 5).

Previous research in LPT infants has demonstrated a relationship between length of hospitalization and amplitude-integrated electroencephalogram (aEEG) findings (4). Neurological function and maturation can also be evaluated by assessment of the autonomic nervous system (ANS) with measures such as heart rate variability (HRV) (6–10). In fact, changes in HRV have been demonstrated to reflect neurophysiological maturation in the neonatal population (11). Advancing GA is accompanied by increasing HRV values, with a power increase most marked in the high frequency (HF) band. As the HF component reflects parasympathetic influence on HR control, this finding suggests a greater maturation of the vagal component during maturation of the ANS (12–15). Therefore, we hypothesized that early postnatal measurement of HRV and aEEG in LPT infants could be a physiological biomarker of their variable clinical trajectories. For that, we examined the associations of HRV and aEEG measured shortly after birth with time to wean to an open-air cot and to time to achieve full oral feeds.

## Methods

### Population

The Neurophysiologic Maturation Index (NEMO) Project (ClinicalTrials.gov Identifier: NCT02156817) was a pilot, international study in stable infants born with GA between 34^0/7^ and 34^6/7^ weeks at three neonatal intensive care units (NICU), namely, McGill University Health Center (Montreal, QC, CA), Hutzel Women's Hospital (Detroit, MI, USA), and Women and Infants Hospital (Providence, RI, USA). GA was assessed by obstetric criteria [presence of a reliable last menstrual period (LMP) date or sonogram performed in the first trimester or agreement between LMP and a sonogram performed between the first trimester and 20 weeks]. Infants were excluded if they had major congenital or genetic anomalies, growth restriction (birth weight <10%, Fenton growth curves), exposure to medications within the preceding 12 h which may affect CNS function (e.g., fentanyl, morphine, and midazolam), seizures, neonatal opioid withdrawal syndrome (NOWS) secondary to *in utero* exposure to opioids or at high risk for development of NOWS, and encephalopathy or were expected to be on mechanical ventilation for the first 96 h after birth. The research ethics board of each institution approved the study, and written informed consent was obtained from parents or legal guardians.

### Study design and data acquisition

Three-lead electrocardiogram (ECG) and cross-cerebral aEEG were recorded simultaneously within the first 96 h of life. ECG was recorded for the first hour, whereas aEEG recordings continued up to 6 h. This was timed in between hands-on or kangaroo care typically after the feeding. ECG leads were placed on the infant's chest or limbs, positioned at least 1 cm apart from the pre-existing leads used for clinical care, and connected to a bioamplifier and PowerLab data acquisition system (ADInstruments, Colorado Springs, CO, USA). The aEEG was obtained using the BRM3 BrainZ Monitor with the Neonatal Hydrogel Sensors (Natus Medical Inc., Pleasanton, CA, USA). Sensors were placed at the C3-P3 and C4-P4 locations on the respective left and right sides of the head with an extra ground sensor placed on the shoulder or between the scapulae.

### Heart rate variability (HRV)

HRV analysis was performed offline using the HRV module of the LabChart (version 2.0, ADInstruments). The last 5 min segment of the ECG that had ≥500 regular intervals (100–200 bpm) was selected independent of the wake/sleep cycle. Time domain, frequency domain, and non-linear HRV parameters were calculated from the segment. The following *time domain* parameters were calculated: standard deviation of the RR intervals (SDRR), standard deviation of heart rate (SD heart rate), coefficient of variation of RR intervals (CVRR), standard deviation of the successive differences between RR intervals (SDSD), and percentage of adjacent RR intervals that differ by greater than 50 ms (pRR50). The following *non-linear* parameters were obtained: standard deviation of intervals perpendicular to the identity line of the Poincaré plot (SD1) and standard deviation of intervals along the identity line (SD2). For the *frequency domain* the following parameters were obtained: TP, total power (<0.4 Hz); VLF, very low frequency (LF) power (<0.04 Hz); LF, low frequency power (0.04–0.15 Hz); HF, high frequency power (>0.15 to <0.4 Hz); and LF/HF ratio, ratio of the low frequency to high frequency power.

### Amplitude-integrated electroencephalography (aEEG)

The scalp was systematically prepared with Nuprep gel to achieve a low impedance. Recordings were stored as digital files for offline analysis. The cross-cerebral aEEG was derived from P3-P4 EEG channels after amplification, filtering, smoothing, and time compression to yield a band of activity which is plotted on a semilogarithmic scale (µV) on the vertical axis and time on the horizontal axis. The voltage values were determined by the BRM3 and analyzed by two investigators (BAS and ARL) using a combined approach of visual and offline digital analysis (BrainZ Analyze software program). Initial visual inspection identified intermittent widening of the band, which was evaluated for the criteria of cycles. These potential cycles allowed preliminary separation of the aEEG tracing into cycles and inter-cycle periods. The Analyze program provided voltages for the upper and lower borders at 1 min intervals throughout the tracing and was exported to a Windows Excel spreadsheet. Voltages were used to determine if visually assessed cycles fulfilled predefined voltage criteria of a cycle and subtypes (mature, immature, and interrupted) based on prior work (4). Cycle frequency was calculated as the number of cycles in the tracing divided by the length of the recording. The percent distribution of the type of cycles was derived for each infant. The inter-cycle period was characterized by an upper and lower border voltage and the difference representing the span, and the percent discontinuity was defined as the percentage time when the lower border voltage is <5 µV.

### Clinical data

Maternal and pregnancy demographics (multiple gestation, gestational hypertension, chronic hypertension, hypothyroidism, preterm labor, length of rupture of membranes, placental abnormality, cord accident, cesarean section, gestational diabetes, antenatal antibiotics, and betamethasone), patient demographics [birth weight, GA, sex, Apgar scores, transient tachypnea of the newborn, respiratory distress syndrome, use of surfactant, delivery room respiratory support, hypoglycemia (<40 mg/dl beyond 4 h of age), highest respiratory support received], and outcome variables (time to wean to an open-air cot and time to achieve full oral feeds) were prospectively collected until discharge or transfer from the units. Local practices were followed for the time to wean to an open-air cot when the infant's temperature remained stable (≥36.5°C/97.7°F) in an ambient temperature of 28°C or less in an incubator for at least 12–24 h, which typically occurred at a weight of approximately 1,800 g, and time to achieve full feeds at 140 cc/kg/day orally at the enrolling centers as per institutional guidelines (16, 17).

### Statistical analysis

Values are presented as median [IQR] or *n* (%). Correlations were evaluated using stepwise linear regression testing with the following model building procedure: first, a subset of physiological variables were preselected by excluding one of variable of pairs with obvious collinearity (i.e., SDRR and SD heart rate); second, univariate linear regressions were assessed between each preselected variable and each outcome; third, variables with regressions at *p* ≤ 0.25 were chosen; fourth, collinearity was assessed within those chosen variables with removal of one of the two variables if strongly correlated (*R* ≥ 0.7); the final variables were tested using stepwise linear regression. Stepwise regression model settings included a *p*-value entry of <0.05 and a *p*-value removal of >0.10 automatically tested toward minimizing the sum of squared errors (MATLAB R2018b, MathWorks, Natick, MA, USA).

## Results

A total of 26 late preterm infants were studied, of which 23 were included for analysis for the outcome of time to an open-air cot (Table 1); three patients were not included due to poor quality or lack of aEEG recordings. The analysis for time to full oral feeds was limited to 19 infants from two centers with similar feeding policies. Infants included for analysis had a median birth weight of 2,140 g (2,045–2,238 g) and GA 34.3 weeks (34.0–34.4 weeks); all patient demographics, maternal and pregnancy demographics, and final outcomes are presented in Table 1. All HRV and aEEG values are summarized in Table 2.

**Table 1:** Clinical characteristics of the study population.

Variables	Values (*n* = 23)
Pregnancy and maternal demographics	
Multiple gestation	6/23 (26)
Gestational hypertension	8/23 (35)
Chronic hypertension	1/23 (4)
Hypothyroidism	1/23 (4)
Preterm labor	19/23 (83)
Length of rupture of membranes (hours)	3.3 [0.5–11.4]
Placenta previa	1/23 (4)
Nuchal cord or knot	10/23 (44)
C-section	9/23 (39)
Gestational diabetes	3/23 (13)
Antenatal antibiotics	15/23 (65)
Antenatal betamethasone	14/23 (61)
Patient demographics	
Birth weight (grams)	2,140 [2,045–2,238]
Gestational age (weeks)	34.3 [34.0–34.4]
Male gender	9/23 (39)
Apgar (1 min)	8 [7–8]
Apgar (5 min)	9 [8–9]
Transient tachypnea of the newborn	3/23 (13)
Respiratory distress syndrome	2/23 (9)
Surfactant	1/23 (4)
Delivery room respiratory support	6/23 (26)
Hypoglycemia	6/23 (26)
Highest respiratory support	
None (room air)	16/23 (70)
Nasal cannula/hood	1/23 (4)
High flow nasal cannula (≥2 L/min)	2/23 (9)
Continuous positive airway pressure	3/23 (13)
Mechanical ventilation	1/23 (4)
Final outcomes	
Time to open-air cot (days)	6 [3–7]
Time to full oral feeds (days)	7 [5–8] (*n* = 19)

Values are presented as median [IQR] or *n* (%).

**Table 2:** Heart rate variability and amplitude-integrated electroencephalography values.

Parameters	Values (*n* = 23)
Heart rate variability	
Time domain	
*R*-wave to *R*-wave interval (ms)	446 [415–464]
SDRR (ms)	27.8 [22.0–35.5]
CVRR	0.066 [0.047–0.077]
Average heart rate (bpm)	134 [130–144]
Standard deviation (SD), heart rate (bpm)	9.2 [6.4–10.2]
SDSD (ms)	10.0 [7.32–15.0]
RMSSD (ms)	10.0 [7.32–15.0]
pRR50 (%)	0.4 [0–1.2]
Non-linear	
SD1 (ms)	7.1 [5.2–10.6]
SD2 (ms)	38.9 [30.0–48.0]
Frequency domain	
Total power (μs^2^)	652.7 [494.9–1,121.5]
Very low frequency (VLF) power (μs^2^)	416.1 [255.3–662.9]
Low frequency (LF) power (μs^2^)	198.0 [143.0–292.5]
High frequency (HF) power (μs^2^)	41.5 [18.5–105.7]
LF/HF ratio	4.9 [2.3–7.2]
Amplitude-integrated electroencephalography	
Cycles	
Total cycles/hour	0.50 [0.35–0.67]
Mature cycles/hour	0.29 [0.17–0.38] (*n* = 19)
Immature cycles/hour	0.29 [0.17–0.33] (*n* = 19)
Interrupted cycles/hour	0 (0, 2.33) (*n* = 19)
Inter-cycle interval	
Average upper border voltage (μV)	20.3 [17.3–21.9] (*n* = 19)
Average lower border voltage (μV)	7.9 [7.6–8.4] (*n* = 19)
Span (μV)	11.7 [10.0–13.5] (*n* = 19)
% Discontinuity	0.3 [0–2.1] (*n* = 19)

Values are presented as median [IQR] or median (min, max); SDSD, standard deviation of the successive differences between RR intervals, RMSSD, root-mean-square of successive differences between RR intervals, pRR50, percentage of RR intervals differing by >50 ms; SD1, standard deviation of points perpendicular to the identity line of the Poincaré plot; SD2, standard deviation of points along the identity line of the Poincaré plot. aEEG parameters as defined by Sommers et al. (4).

**Figure 1:**
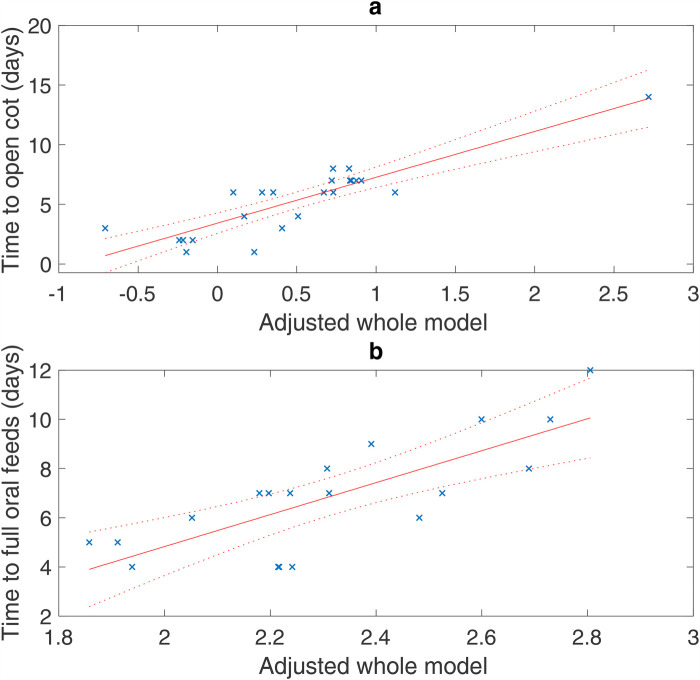
Regression models of all adjusted data (×) with the 95% confidence bounds (dotted line). *X*-axis: adjusted model is a single-value representation of the multiple variables and their coefficients for each patient included in the model, e.g., adjusted model = [b1 × 1 + b2 × 2 + b3 × 3 +…]. **(a)** Time to an open-air cot: *R*^2^ = 0.720 adjusted for SDRR, LF/HF ratio, interaction between SDRR and LF/HF ratio, and total cycles/hour (*p* < 0.001). **(b)** Time to full oral feeds: *R*^2^ = 0.527 adjusted for median RR interval, LF/HF ratio, immature cycles/hour (*p* = 0.002).

The details of the regression models for both outcomes are provided in Tables 3 and 4. For both outcomes, the best model was found with both HRV and aEEG variables (Table 4). The model for time to an open-air cot included HRV parameters SDRR and LF/HF ratio, an interaction term between SDRR and LF/HF ratio, and the aEEG parameter total cycles/hour. The time to an open-air cot model is strong, with an adjusted *R*^2^ of 0.720 (Table 4; Figure 1a). Similarly, for the outcome of time to full oral feeds, the best model included median RR and LF/HF ratio HRV parameters, with the immature cycles/hour aEEG parameter (Table 4). This model has modest strength, with an adjusted *R*^2^ of 0.527 (Table 4; Figure 1b).

**Table 3:** Multivariate regression analysis of outcomes influencing length of hospital stay with HRV and aEEG parameters separately.

Model parameters	Coefficient (B)	95% CI	*p*-value	Adjusted *R*^2^
Time to an open-air cot (days), *n* = 23				
Model: HRV parameters				
Intercept	7.247	4.482–10.013	<0.001	0.704
SDRR	−0.700	−1.072 to −0.328	0.002	
VLF power	0.004	0.002–0.006	0.012	
LF/HF ratio	0.448	0.252–0.644	<0.001	
Model: aEEG parameters				
Intercept	2.897	0.570–5.224	0.024	0.169
Total cycles/hour	5.199	0.846–9.551	0.029	
Time to full oral feeds (days), *n* = 19				
Model: HRV parameters				
Intercept	11.245	8.655–13.835	<0.001	0.366
Median RR	−0.721	−1.150 to −0.292	0.005	
LF/HF power	0.004	0.001–0.007	0.024	
Model: aEEG parameters				
Intercept	4.872	3.122–6.621	<0.001	0.238
Immature cycles/hour	7.527	1.794–13.260	0.020	

HRV, heart rate variability; aEEG, amplitude-integrated electroencephalography; SDRR, standard deviation of RR intervals; VLF, very low frequency; LF/HF, low frequency/high frequency; RR, *R*-wave to *R*-wave interval.

**Table 4:** Stepwise regression models for clinically important outcomes integrating physiological variables of HRV and aEEG.

Model parameters	Coefficient (B)	95% CI	*p*-value	Adjusted *R*^2^
Time to an open-air cot (days), *n* = 23				
Intercept	3.425	0.269–6.581	0.035	0.720
SDRR	−0.040	−0.112 to 0.032	0.257	
LF/HF ratio	0.613	0.285–0.941	0.001	
SDRR × LF/HF ratio	−0.015	−0.028 to −0.002	0.027	
Total cycles/hour	3.792	0.268–5.263	0.012	
Time to full oral feeds (days), *n* = 19				
Intercept	−8.162	−18.486 to 2.162	0.113	0.527
Median RR	0.026	0.003–0.049	0.030	
LF/HF ratio	0.303	0.088–0.519	0.009	
Immature cycles/hour	6.489	1.344–11.633	0.017	

HRV, heart rate variability; aEEG, amplitude-integrated electroencephalography; SDRR, standard deviation of RR intervals; LF/HF, low frequency/high frequency; SDRR × LF/HF ratio, interaction between SDRR and LF/HF ratio; RR, *R*-wave to *R*-wave interval.

## Discussion

This pilot prospective observational study of LPT infants explored possible associations between HRV and aEEG measured shortly after birth, and subsequent time to reach important clinical milestones required for safe discharge from the NICU. The combination of the HRV and aEEG variables has a stronger effect on the subsequent time to achieve full oral feeds given a substantial increase in *R*^2^ when both variables are included. In contrast, HRV alone has an *R*^2^ of 0.70 for time to an open-air cot and goes up by a small amount when combined with aEEG.

### HRV

Increases in HRV are described with increasing GA and post-conceptional age (18–21). Specifically, increases in LF and HF power have been observed (18–21) with slight decreases in the LF/HF ratio (18), indicating an unequal rise, and increases in time domain parameters (21). With these changes, higher values in time domain and frequency domain parameters (more mature) would be associated with shorter times of incubator weaning times and to achieve full oral feeds; thus, negative coefficients should be observed. Our results are in line with these maturational changes, as a negative correlation was observed with the time domain parameter SDRR. Moreover, a positive correlation with both outcomes and the LF/HF ratio was noted, as the LF/HF ratio decreases with maturation (18).

A positive correlation was also observed with the median RR interval for the time to full oral feeds outcome; this, however, was not expected given that the RR interval should typically increase, equivalent to heart rate decreasing, with maturation (18, 21). However, this finding may suggest an indication of better adaptability within this narrow range of GA. A maturational lag in vagal function may contribute to feeding difficulties in LPT infants (22). Moreover, preterm infants often have an underdeveloped parasympathetic nervous system, which could impair their response to feeding (23). This impaired parasympathetic response could potentially correlate with longer median RR intervals and delayed feeding progression. Since this was a pilot study with limited power, this hypothesis-generating unexpected observation warrants further study to advance our understanding of the neurophysiological maturation of preterm infants.

### aEEG

Distinct changes in aEEG have been demonstrated across GAs, characterized by increasing presence of continuous activity and the development of sleep–wake cyclic changes with maturity (24, 25). Furthermore, Sommers et al. (4) examined the aEEG sleep–wake cycles in a cohort of LPT infants within a narrow GA of 34^0/7^–34^6/7^ weeks as in this study with variations in cycling, which was inversely correlated with the length of hospital stay. Our results are consistent with a previous study as inclusion of aEEG variables improved the correlation between HRV and clinical determinants of length of hospital stay.

This association was robust for time to an open-air cot compared with time to achieve full oral feeds. Successful oral feeding is a complex neurologic milestone as it requires the integration and coordination of oropharyngeal, respiratory, and gastrointestinal systems, as well as maturation of sensory systems (visual, auditory, somatosensory, gustatory, and olfactory) and hypothalamic pathways which encode satiety and hunger (26). Additionally, unlike the stabilization of suck and swallow rhythms, which occur before 36 weeks' GA, improvement in coordination of respiration and swallow begins later (27, 28). These biological complexities may contribute to the limited strength of the correlation for time to achieve full oral feeds. Further research is warranted to advance our understanding of the differential developmental maturation of the parasympathetic and sympathetic nervous systems in LPT infants.

### Limitations

This study has some limitations. First, there are variations in clinical practice across sites (e.g., timing of cot weaning and feeding advancement) as none of the involved sites had established policies on when to transfer LPT infants from a closed incubator to an open-air cot and advancing oral feeds. Weaning often took place subjectively, based on general thermoregulatory stability as established by minimal fluctuations in incubator temperature, the level of respiratory support, and infants' weight of typically ≥1,800g. It is unclear what kind of effect this may have had on the data. We suggest standardization of the protocol for these clinical milestones in future studies to control for any potential bias. Second, the study was stopped due to a change in the affiliation of the investigator resulting in a small sample size, which may limit the generalizability and the robustness of regression models, and a larger sample size would permit the introduction of clinical variables. Third, 23 infants had usable aEEG data, and 3 infants were excluded due to lack or suboptimal quality signals with high impedance (>5 kΩ). Additionally, four infants from one site could not have their aEEGs analyzed beyond the total cycles/hour due to incompatibility of file formats. Finally, the median age at time of physiological recording was 71 h [IQR, 48–78 h], and the median time to open-air cot was 6 days [IQR, 3–7 days]. Hence, in some cases, recordings may have been done after cot transition. We therefore advise caution in interpretations regarding the predictive ability of this regression.

## Conclusion

HRV and aEEG parameters measured shortly after birth correlated with subsequent time to an open-air cot and to achieve full oral feeds in infants born between 34^0/7^ and 34^6/7^ weeks GA. Both parameters provided complementary information regarding the neurophysiological maturation. These results support the hypothesis that differences in ANS function may contribute to the variability in the time to reach milestones for safe discharge among LPT infants at the same GA. Future studies with larger sample sizes are needed to confirm these preliminary observations, which would further allow the addition of other variables and better understand the potential of these physiological measurements as biomarkers of clinical trajectories.

## Data Availability

The raw data supporting the conclusions of this article will be made available by the authors, without undue reservation.
